# Caleb Parry of Bath

**Published:** 1956-01

**Authors:** John Apley

**Affiliations:** Consultant Paediatrician Bristol Children's Hospital and Royal United Hospital, Bath


### CALEB PARRY OF BATH

*The bi-centenary of a great West Country physician*

BY

JOHN APLEY

*Consultant Paediatrician Bristol Children's Hospital and Royal United Hospital, Bath*

Nobody, so far as I know, has collated and set down the achievements of physicians from the West Country, though they form a galaxy as bright as any. Our only claim to some of these great men is that they were born in this part of England; but it was in the West Country that others, and not the least distinguished, practised, pioneered and made their names.

The best known is surely Edward Jenner (1749–1823),* of vaccination fame, who was born and later practised in Gloucester. Believing what a milkmaid told him, he transmuted a local bucolic tradition into a universal scientific principle. Less known, and perhaps more of a Pandora's box, is his early description and discussion of allergy. Jenner's *An Inquiry into the Causes and Effects of the Variolae Vaccinae* was dedicated to " C. H. Parry, M.D., at Bath, My Dear Friend ", who is the main subject of this essay; but, before turning to him, a few other brilliant names may scintillate before us for a moment.

In Bristol was born Richard Bright (1789–1858) who became one of " The great men of Guy's ". He sprang to fame with his epoch-making distinction between cardiac and renal dropsy. " Bright could not theorize," said Wilks " but he could see "; and we read with admiration of the many and varied conditions he observed, often before anybody else. They include pancreatic diabetes, pancreatic steatorrhoea, acute yellow atrophy of the liver, cerebral hemiplegia, echinococci in hydatid cysts and cardiac murmurs in chorea. A little before Bright's time was Thomas Baynton (1761–1830), a Bristol surgeon, the first to treat spinal caries by absolute rest in the horizontal position. Francis Glisson (1597–1677) was born in Rampisham in Dorset; his name is best known for " Glisson's Capsule " of the liver, but his great achievement was the first full account of rickets in infants. Two Cornishmen were Richard Lower (1631–91), the first to perform direct transfusion of blood from one animal to another, and John Mayow (1643–79), a genius of physiology. In his short life he conceived the idea, and demonstrated by experiments, that the object of breathing is to produce an interchange of gases between the air and the blood, shown by corresponding changes in the colour of the blood from dark to bright red. This antechronistic idea was conceived before Lavoisier, in 1789, had demonstrated that the respiration of living organisms involves oxygen. Among Devon men there were James Yonge (1646–1721), surgeon to the naval hospital at Plymouth, whose book extolling the virtues of turpentine, especially in the control of haemorrhage, can be seen in Bristol University Medical Library; John Huxham (1692–1768) of Totnes, the first to observe palatal paralysis in diphtheria; and Sir George Baker (1722–1809), who antagonized his fellow Devonians by (correctly) attributing Devon " cider-time colic " to lead. And at Exeter was John Sheldon (1752–1808), who lectured under the renowned William Hunter in London, followed him as professor of anatomy at the Royal Academy, and eventually came as surgeon from the Westminster Hospital to the Devon and Exeter Hospital, where his portrait hangs now. As Mr. Norman Capener has told us, " Sheldon's interests in embalming, lymphatics, whaling and ballooning make up a story of great fascination that

* Not to be confused with Sir William Jenner (1815–1898), physician to Queen Victoria, who helped differentiate typhus and typhoid, one of whose sons, Colonel L. C. D. Jenner, died only two years ago at 9 Circus, Bath.

must remain for another occasion", and we must hope that Mr. Capener will record it for our further enjoyment. The name of Thomas Willis (1621–75), son of a Wiltshire farmer, is remembered by the "Circle of Willis"; but his was also the first description of myasthenia gravis, and his the original observation of the sweet taste of diabetic urine.

Eminent among West Country physicians, many of whom we must pass by in the Hall of Fame, is Caleb Hillier Parry (1755–1822). Sixteen years after Jenner had dedicated his now classic work on vaccination to Parry, the compliment was returned when Parry dedicated a work on tetanus and rabies to Jenner, his "dear and oldest friend". The friendship between these two great men, which was to continue throughout their lives, had commenced when they attended the same school in Cirencester, the town in which Parry was born. But Parry's name is associated with the City of Bath, to which he moved in 1779 with his bride, and from which he hardly travelled during the remaining forty years of his life. Shortly after settling in Bath Parry became physician to the Casualty Hospital which was merged with the City Infirmary in 1826; they formed the United Hospital,* whose first physician was Parry's eldest son. Parry later became physician also to the Bath General Hospital, now so widely known as the Royal Mineral Water Hospital.

In Bath, Parry lived first at 13 Catherine Place. From there he moved round the corner to 27 Circus, in a centre so preponderantly medical now that, like the Harley Street area in London, it is referred to by taxi-drivers as "The Pill Box". The last years of his life were spent, still actively concerned with research—by now he was carrying out vivisection on sheep—in a house which he had built on Sion Hill.†

Before settling in Bath, Parry, blossoming early, had enjoyed a most distinguished career as a student in Edinburgh. Indeed, under his presidency the Students' Society obtained the still unique distinction for a students' body of a royal charter. Since Parry's day the migration south from Edinburgh to Bath has been repeated by many able doctors.

Parry is justly credited with the earliest recognition of exophthalmic goitre: he described eight cases, under the title *Enlargement of the Thyroid Gland in connection with Enlargement or Palpitation of the Heart.* In *An Introduction to the History of Medicine* (1929) Garrison rightly affirms that this account of exophthalmic goitre was "so complete and original that it more justly entitled him to the honour of its discovery than either Flajani (1800), Graves (1835), or Basedow (1840)". Parry's story and achievements have been delightfully sketched by Sir Humphry Rolleston in the *Annals of Medical History* (1925); but his claim to fame was first spread through the great Osler's advocacy, and exophthalmic goitre was dubbed "Parry's disease" in Osler's *Principles and Practice of Medicine.* To Parry have also been ascribed the first descriptions of congenital megacolon (some sixty or more years before Hirschsprung's description in 1888), facial hemiatrophy, and what is now sometimes termed "histaminic cephalgia". So painstaking and meticulous were his notes that, in *Collections from the unpublished Medical Writings*, edited by Parry's son, descriptions of many previously unrecorded disorders can be recognized. Parry was careful to record observations on his cases on the day they were made. Moreover, he "eagerly availed" himself of "dissection" of the body after death, and his autopsy records read as lucidly as his clinical notes.

Some of his descriptions can serve as models today. Thus: "A physician, of fair complexion, large, fat and of a full habit, at twenty-two became extremely dyspeptic . . . was obliged to leave off fermented liquors." Again, "William B., aged three, had last summer the natural small-pox in a very violent degree, but recovered and

* Mr. Charles Kindersley, doyen of the hospital at the present time, reminds me that in the reign of Queen Victoria the title *Royal* was added and, as far as proved possible, each of the wards was named after one of the royal children.

† In *Georgian Buildings of Bath* Walter Ison notes that the famous John Eveleigh charged twenty guineas for designing and supervising the building, which unfortunately no longer exists.

has enjoyed good health till about four months ago, when he began to complain of a pain in the belly; and had sickness and vomiting, which occurred only in the morning, just after he rose from his bed at six or seven o'clock. Two months ago he had a stiff neck, with giddiness, strabismus, and occasional headache." From his crisp words the workings of Parry's mind can be seen crystal clear. Thus, under the heading "*Squinting cured*": "Master S., aged one year, . . . was perceived, after a violent fit of crying, to squint with the left eye, which turned preternaturally inwards . . . . I recommended that the eye which was well should be constantly covered during the day . . . . His eye regained its proper power, which . . . it continues to possess." Nor is Parry fearful of criticism: "The most dangerous state . . . human mind is a calm acquiescence in the accuracy and extent of its own attainments." He did not shrink from attacking the famous figures of the day if he conceived that they were in error: "When John Hunter speaks of the blood coagulating by the stimulus of necessity, he mistakes the final cause for the . . . means."

The talents of this remarkable man, like those of so many doctors of that period, were by no means canalized in medical channels. He was a Fellow of the Royal Society, became a member of the Society of Natural History of Göttingen, and was made an honorary member of the Farming Society of Ireland for his services to the wool industry. His wide extra-medical interests are shown also by the stimulating variety of his publications and addresses. He wrote several papers in the *Farmers Journal*; lectured on many occasions to the West of England Society of Agriculture, Arts, Manufactures and Commerce; and his published dissertations embrace fossils, wool production, and sheep breeding.

In the medical library of Bristol University reposes a collection of over 900 books, "The Parry Library", lately transferred from its original home in Bath. It includes the "Parry Collections", edited by his son and printed in 1825; the remaining books forming a varied and, indeed, rather odd miscellany of medical and near-medical matters. As might be expected, from a city which now bears with a railway station flaunting the ovine-sounding title "Bath Spa", some are on the subject of "the waters". There is the *Narrative of the efficacy of Bath waters in various kinds of paralytic disorders;* and *Bath Memoirs, or Observations in three and forty years' practice at the Bath: what cures have been there wrought* (1697). There are commentaries, not only on spa waters, but on vapour bathing and sea water. One finds medical books in Greek, Latin, French and German as well as many translations; and there is a book, written in 1646, on Egyptian medicine and Indian medicine. There are many old books on anatomy, some most beautifully engraved, and on surgery "proper not only for the improvement of all young surgeons, but also for the direction of such as are further advanced". An "Account of a child with a double head" set me searching, with the possibility of writing to Professor Ian Aird before me, until I found that the drawings had been done in 1790 and as far from home as Bengal. Not all is materialistic, however, and I draw attention to *A theologico-philosophical dissertation concerning worms in all parts of human bodies* (1727), and *A dissertation on the influence of the passions upon disorders of the body* (1788), though I failed to find the word *psychosomatic* therein. Even for Distinction Awards there is a possible precursor in the *Debates in Parliament respecting the Jennerian discovery, including the late debate on the further grant of twenty thousand pounds to Dr. Jenner.*

Mr. A. E. S. Roberts, of the University Medical Library, who has courteously provided some references and items of interest for this essay, draws my attention to the curious paucity of Parry's own signatures in the books of the Parry Library. Probably we shall never discover a reason for this extraordinary omission; for in our day the Parry Library and the man who inspired it are regrettably neglected. We are rich but forgetful heirs to many great physicians who practised before us in this fruitful corner of our land. Were we to contribute further accounts of their lives and times, and old and interesting medical books, we might set growing again the Parry Library—to the glory of Caleb Hillier Parry and English Medicine itself.

